# Supplementary material for: Synthesis of Substituted 1,2,4-Triazole-3-Thione Nucleosides Using E. coli Purine Nucleoside Phosphorylase
Source: Biomolecules. 2024 Jun 24;14(7):745. doi: 10.3390/biom14070745 (PMC11274511; doi:10.3390/biom14070745)
Supplement: Supplementary file 1 [file biomolecules-14-00745-s001.zip › biomolecules-3029566-supplementary.pdf]

Supplementary Materials

Synthesis of Substituted 1,2,4-Triazole-3-thione Nucleosides Using *E. coli* Purine Nucleoside Phosphorylase

Ilya V. Fateev [1],*, Sobirdjan A. Sasmakov [2],*, Jaloliddin M. Abdurakhmanov [2], Abdukhakim A. Ziyaev [2], Shukhrat Sh. Khasanov [2], Farkhod B. Eshboev [2], Oybek N. Ashirov [2], Valeriya D. Frolova [1], Barbara Z. Eletskaya [1], Olga S. Smirnova [1], Maria Ya. Berzina [1], Alexandra O. Arnautova [1], Yulia A. Abramchik [1], Maria A. Kostromina [1], Alexey L. Kaushin [1], Konstantin V. Antonov [1], Alexander S. Paramonov [1], Valeria L. Andronova [3], Georgiy A. Galegov [3], Roman S. Esipov [1], Shakhnoz S. Azimova [2], Anatoly I. Miroshnikov [1] and Irina D. Konstantinova [1]

[1] Shemyakin and Ovchinnikov Institute of Bioorganic Chemistry, Russian Academy of Sciences, Miklukho-Maklaya St. 16/10, 117997 GSP, Moscow B-437, Russian Federation

[2] Acad. S.Yu. Yunusov Institute of the Chemistry of Plant Substances, Academy of Sciences of the Republic of Uzbekistan, Mirzo Ulugbek Str. 77, 100170, Tashkent, Uzbekistan

[3] D. I. Ivanovsky Institute of Virology (N. F. Gamaleya Research Center of Epidemiology and Microbiology, Ministry of Healthcare of the Russian Federation), Gamaleya St. 18, 123098, Moscow, Russian Federation

* Correspondence: ifateev@gmail.com (I.V.F.); sasmakov@web.de (S.A.S.)

Table of Contents

1 – Octanol/water partition coefficient (logP).

2 – The relationship between lipophilicity logP and cytotoxic properties CC_{50}, antiviral activity IC_{50} and selectivity index SI of compounds **3–13** and ribavirin.

3 – The relationship between lipophilicity logP and cytotoxic properties CC_{50} and antiviral activity IC_{50} of compounds **8-13** and ribavirin.

4 – The NMR spectra of the target products.

Table S1. Octanol/water partition coefficient (logP).

Compound	LogP
**3**	0.18
**4**	2.19
**5**	2.03
**6**	4.59
**7**	4.92
**8**	0.22
**9**	1.04
**10**	3.26
**11**	4.08
**12**	3.58
**13**	4.4
Ribavirin	-1.85

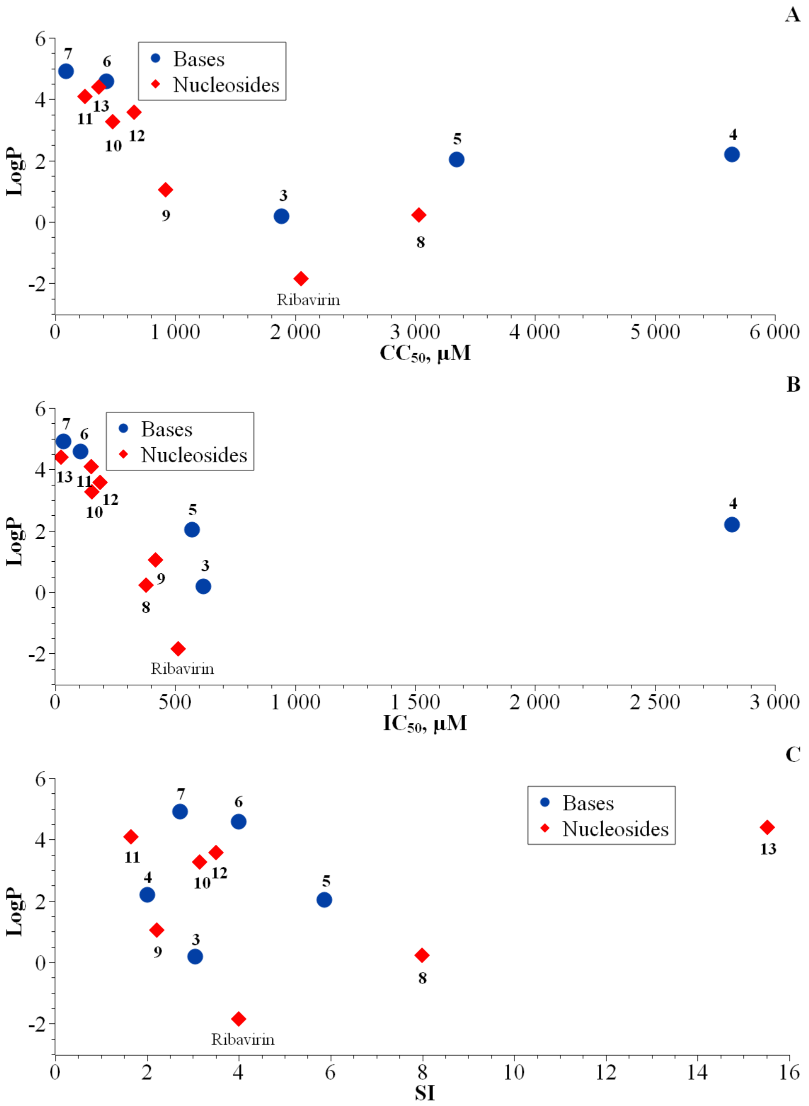

Figure S1. The relationship between lipophilicity logP and cytotoxic properties CC_{50} (A), antiviral activity IC_{50} (B) and selectivity index SI (C) of compounds **3–13** and ribavirin. Data of IC_{50} and SI for strain HSV-1/L2 (TK+). The number of compound is shown next to the dot.

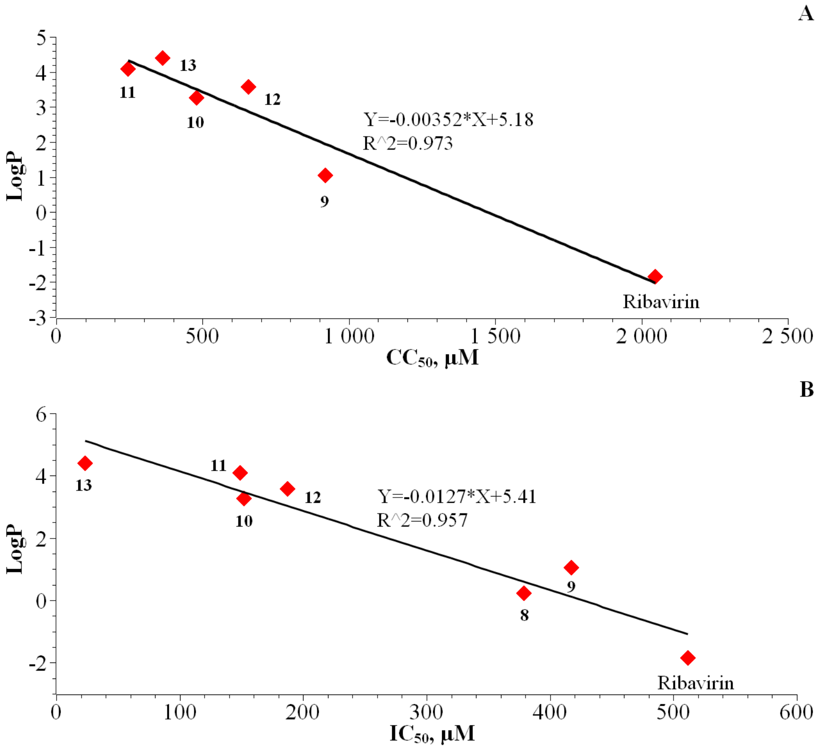

Figure S2. The relationship between lipophilicity logP and cytotoxic properties CC_{50} (A) and antiviral activity IC_{50} (B) of compounds **8-13** and ribavirin. Data of IC_{50} for strain HSV-1/L2 (TK+). The number of compound is shown next to the dot.

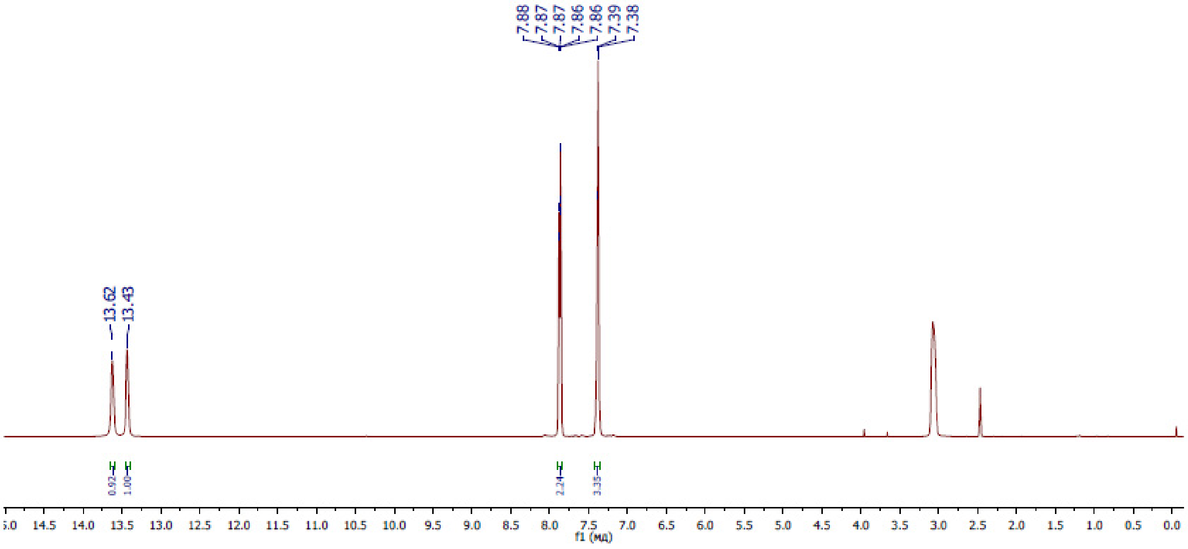

Figure S3: The ^{1}H NMR spectrum of 5-phenyl-1,2,4-triazole-3-thione (**4**)

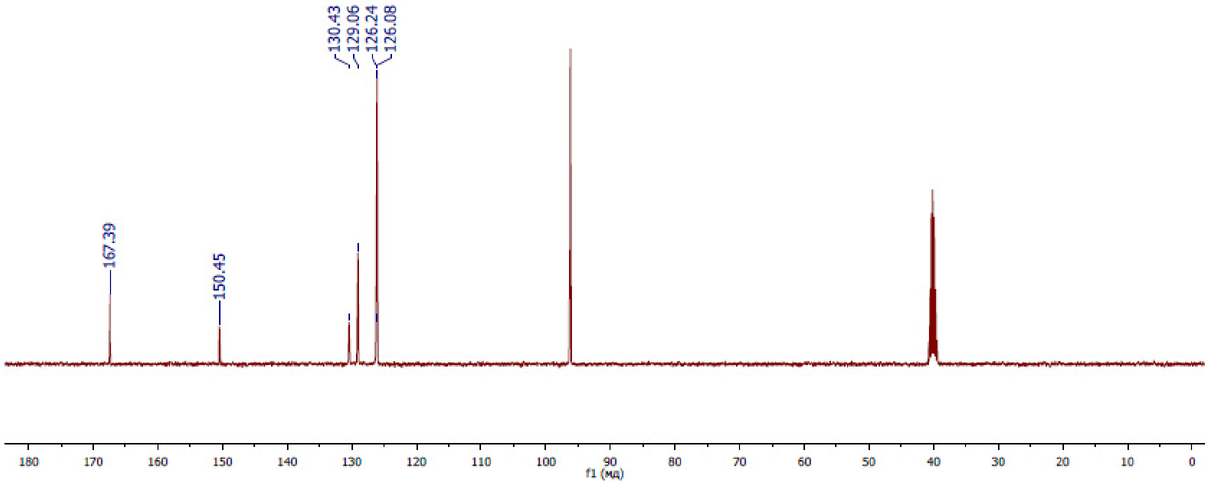

Figure S4: The ^{13}C NMR spectrum of 5-phenyl-1,2,4-triazole-3-thione (**4**)

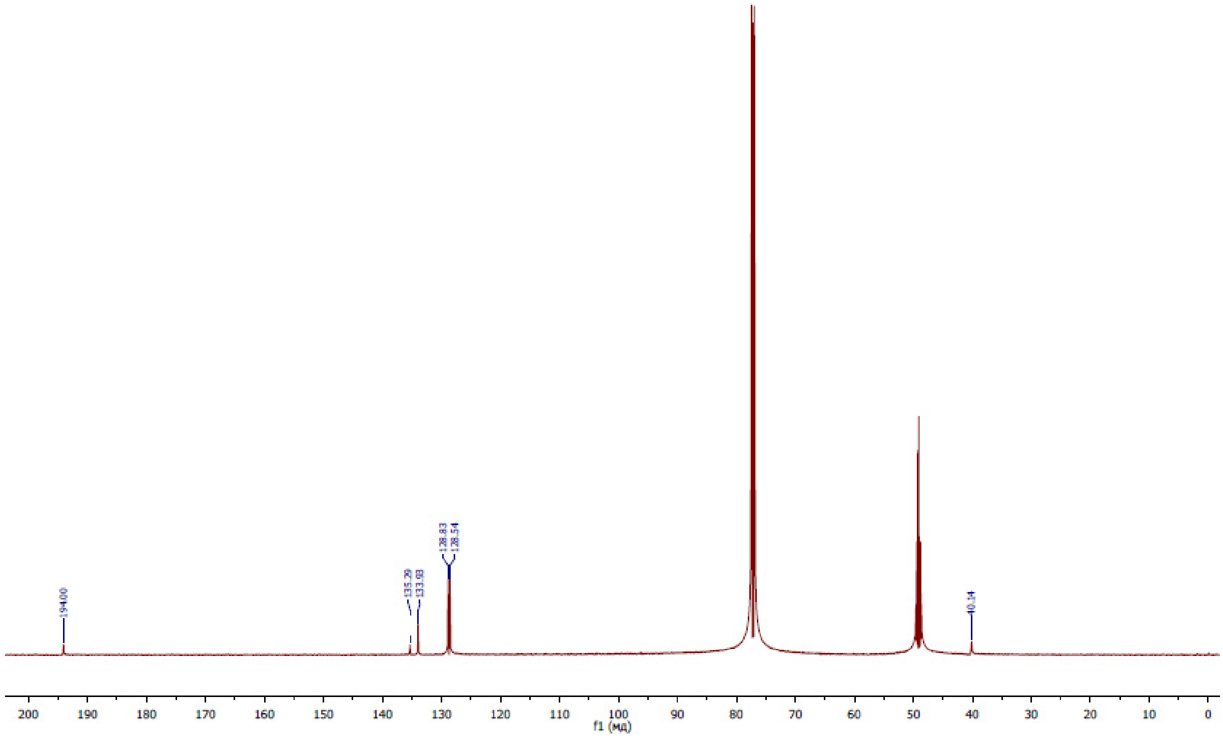

Figure S5: The ^{13}C NMR spectrum of 3-phenacylthio-1,2,4-triazole (**5**)

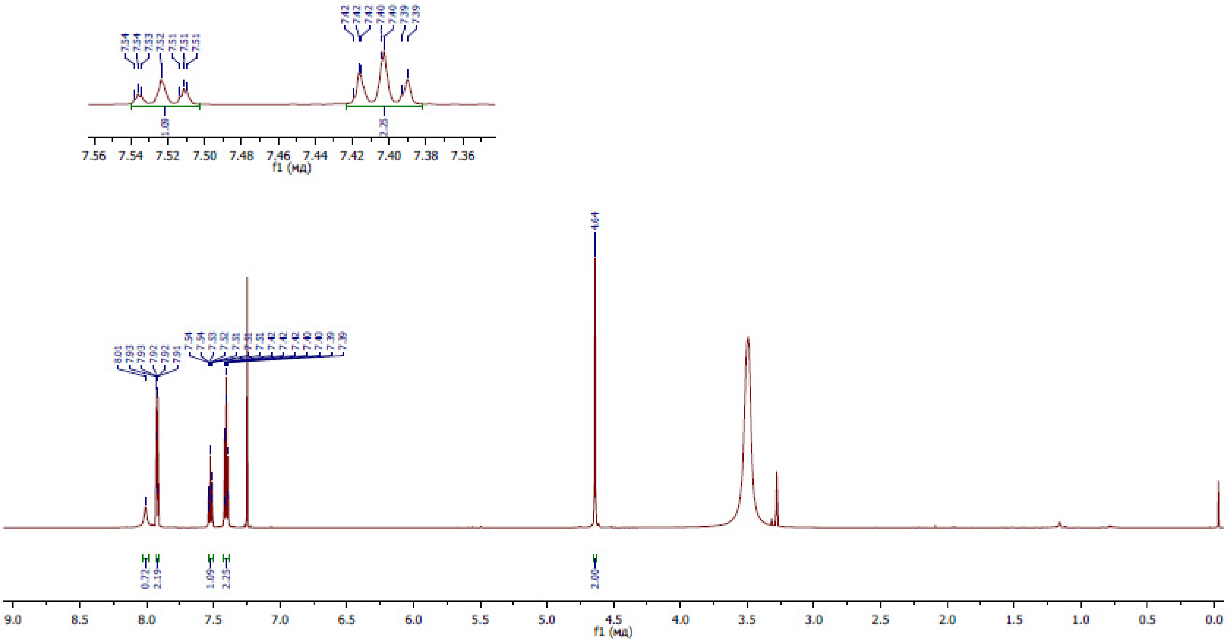

Figure S6: The ^{1}H NMR spectrum of 3-phenacylthio-5-phenyl-1,2,4-triazole (**6**)

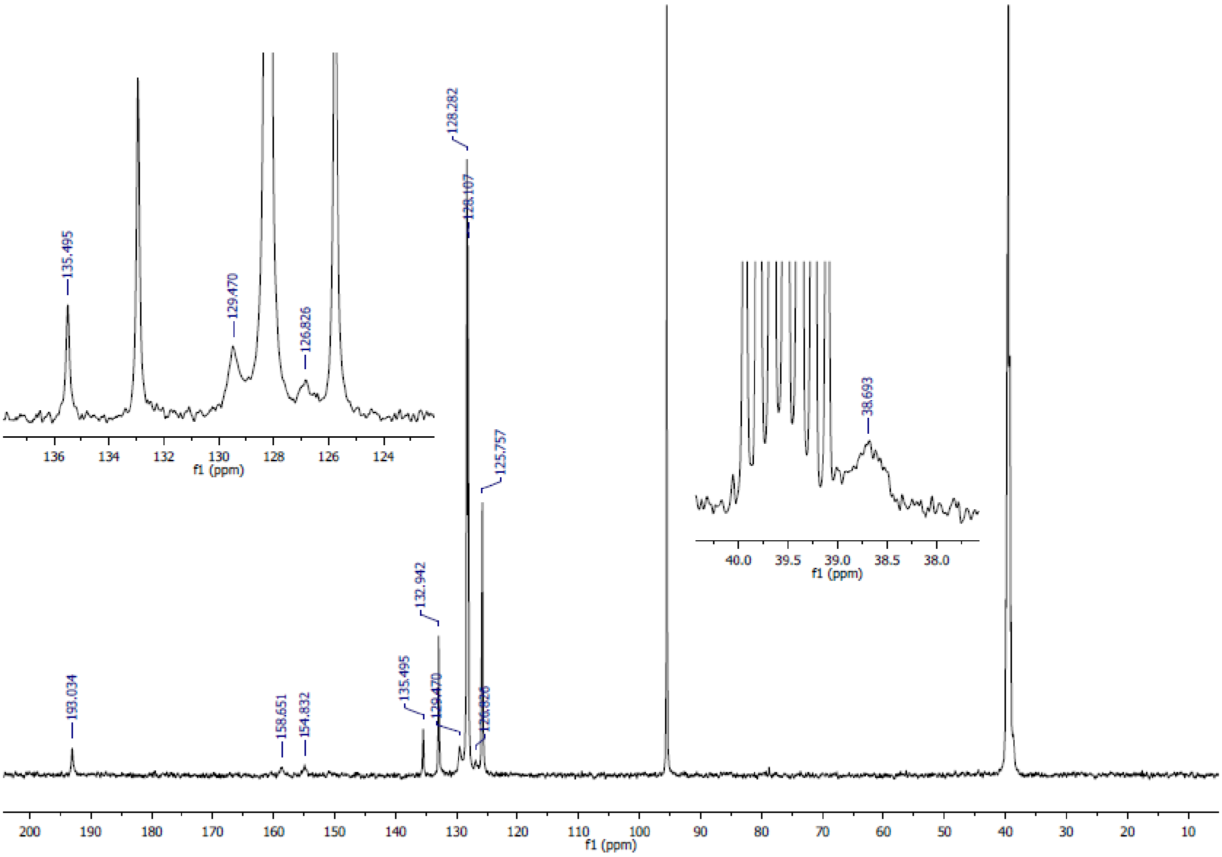

Figure S7: The ^{13}C NMR spectrum of 3-phenacylthio-5-phenyl-1,2,4-triazole (**6**)

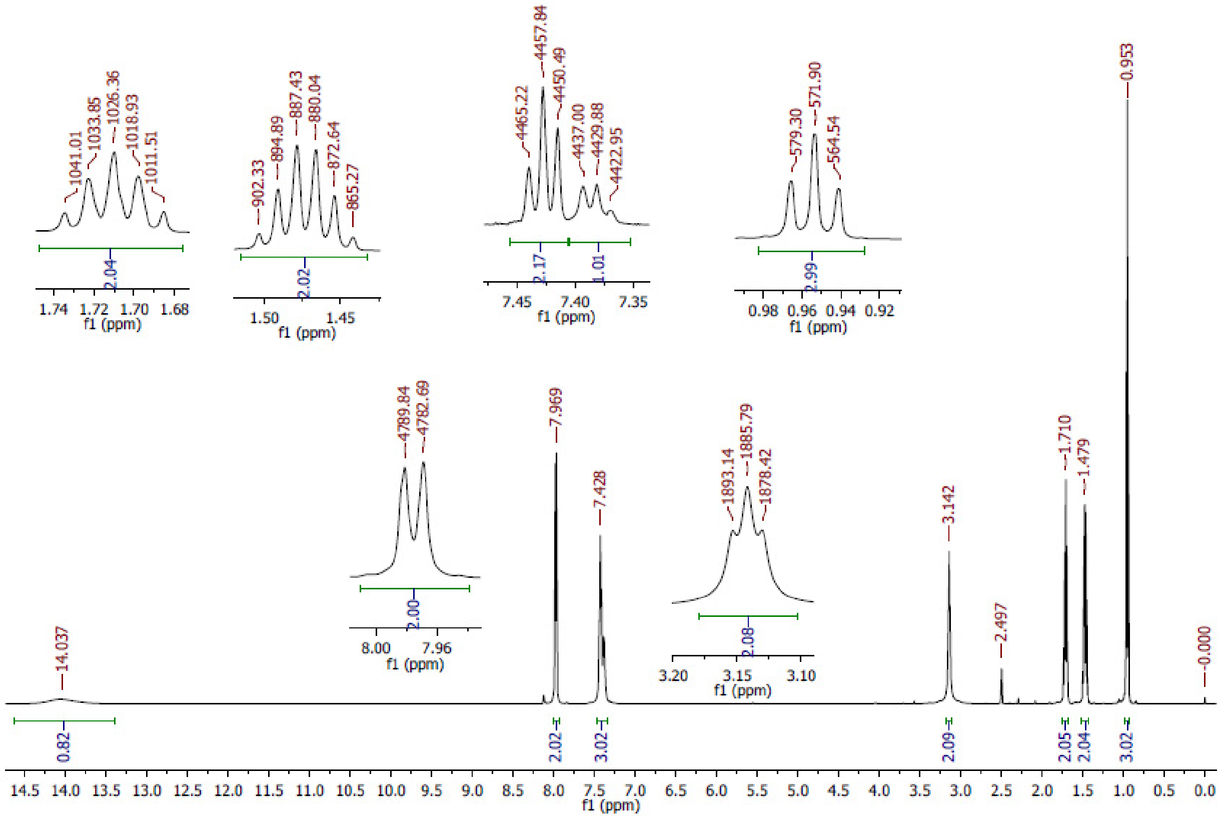

Figure S8: The ^{1}H NMR spectrum of 3-butylthio-5-phenyl-1,2,4-triazole (**7**)

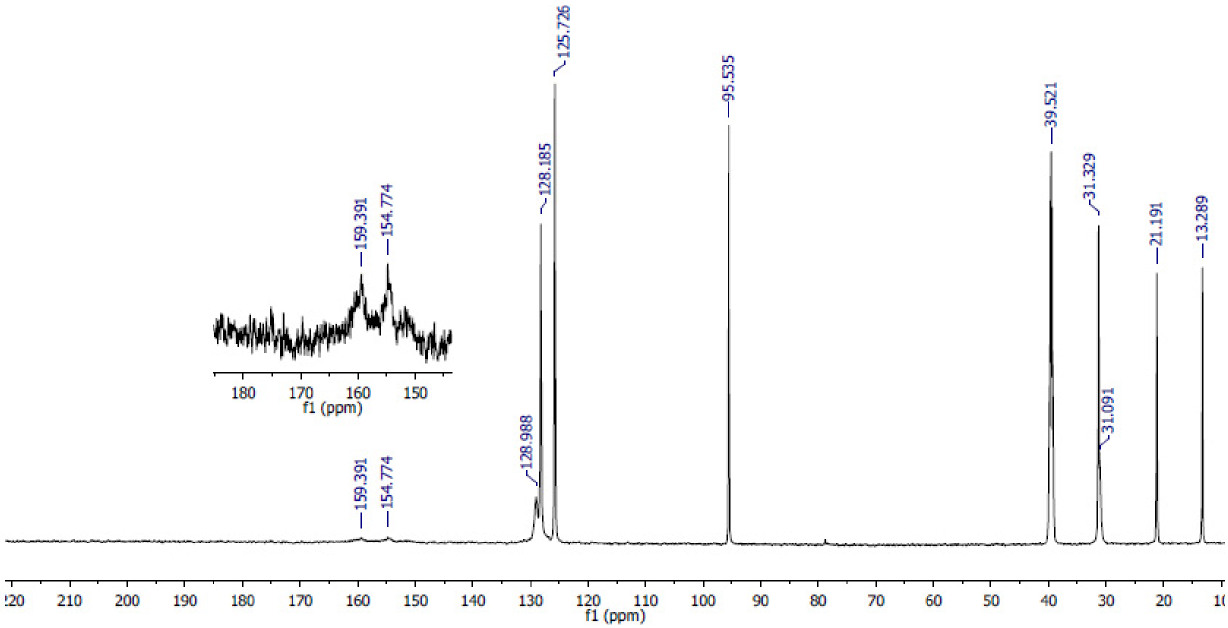

Figure S9: The ^{13}C NMR spectrum of 3-butylthio-5-phenyl-1,2,4-triazole (**7**)

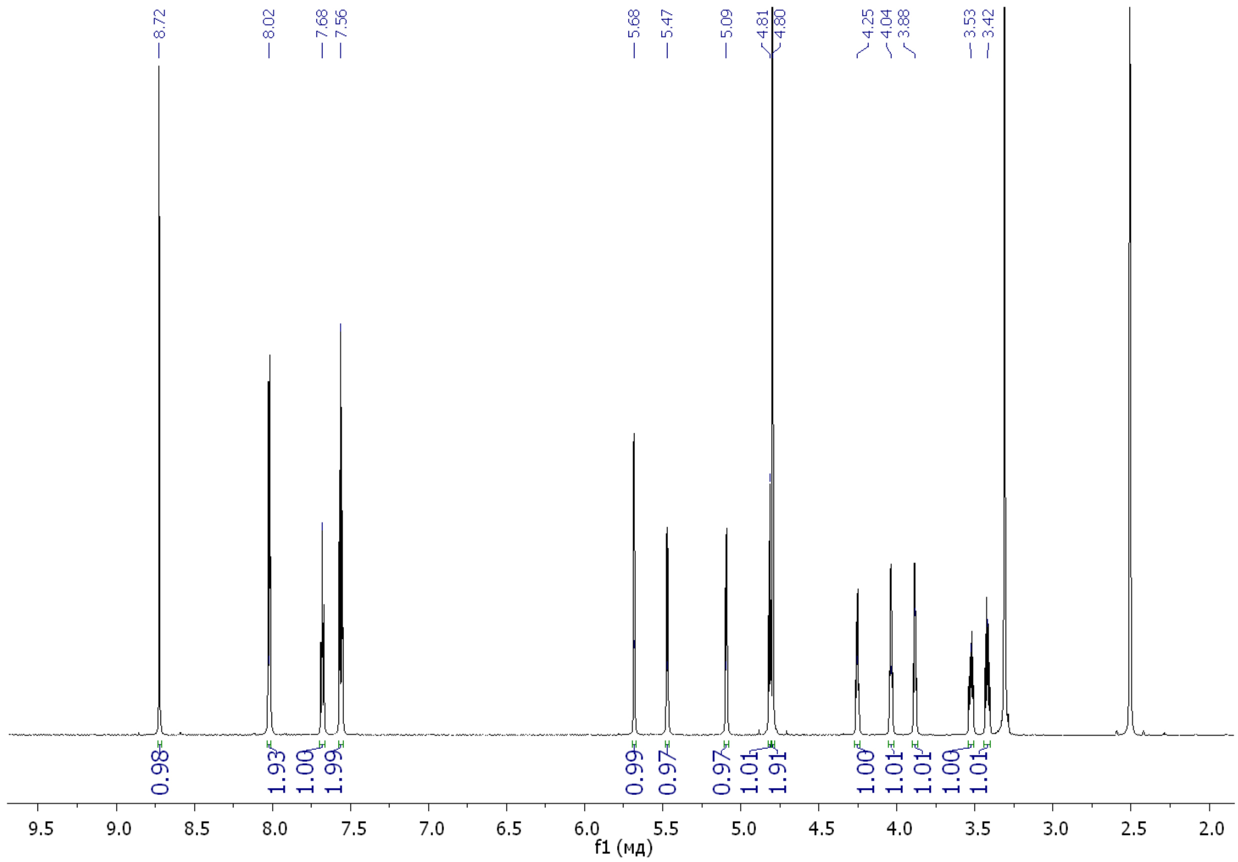

Figure S10: The ^{1}H NMR spectrum of 3-phenacylthio-1-(β-*D*-ribofuranosyl)-1,2,4-triazole (**8**)

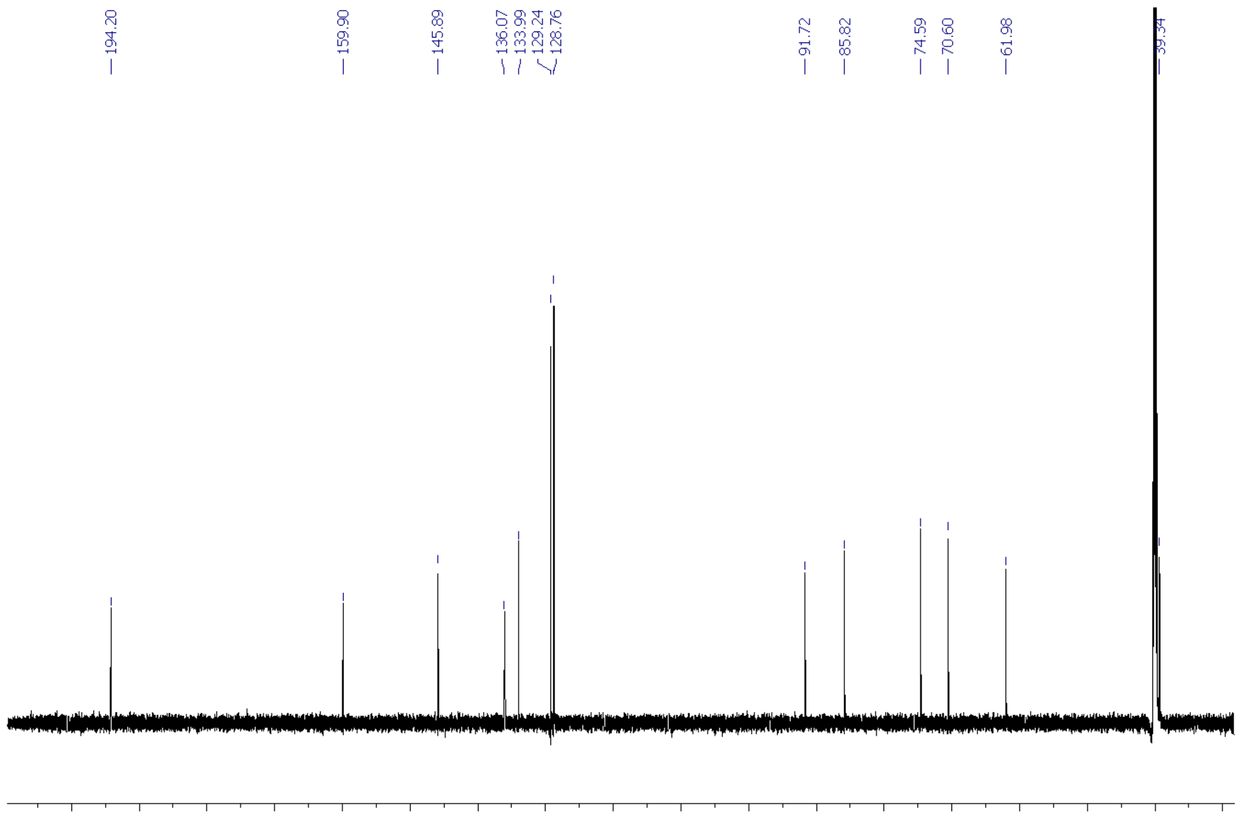

Figure S11: The ^{13}C NMR spectrum of 3-phenacylthio-1-(β-*D*-ribofuranosyl)-1,2,4-triazole (**8**)

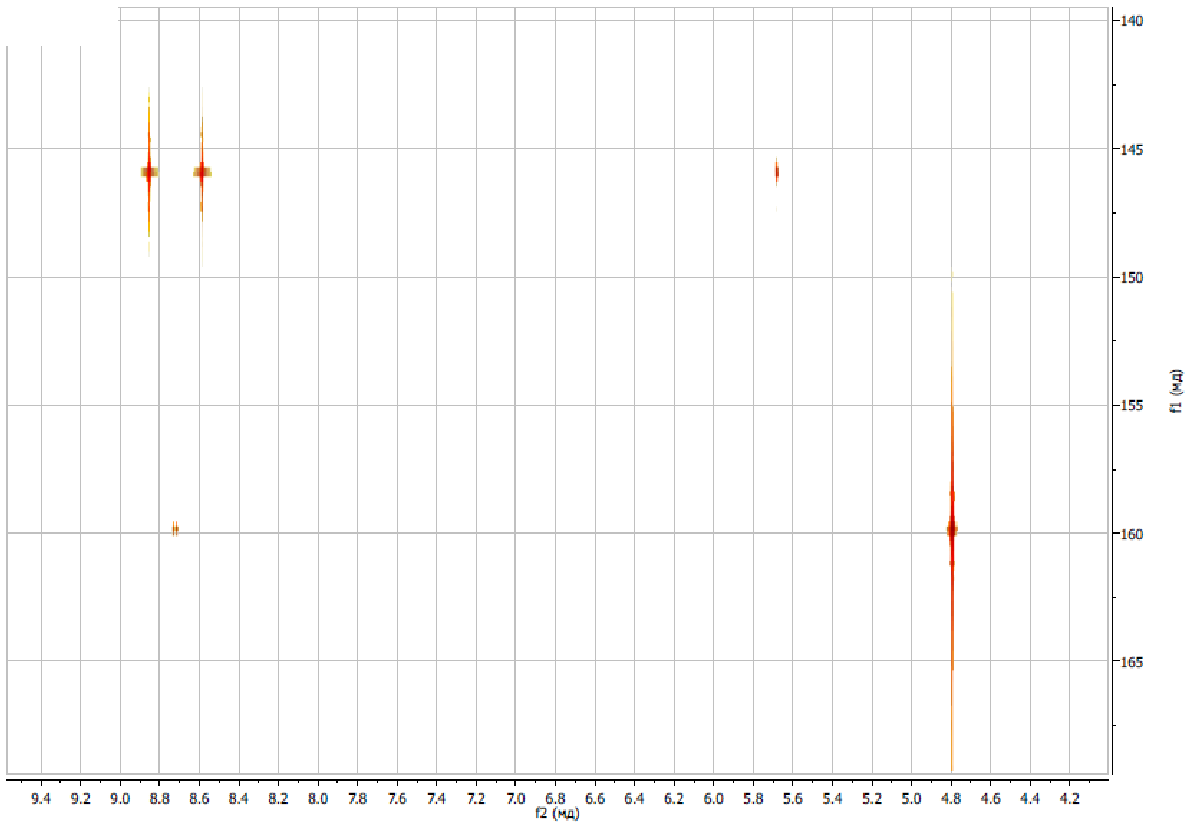

Figure S12: Fragment of the ^{1}H-^{13}C HMBC NMR spectrum of 3-phenacylthio-1-(β-*D*-ribofuranosyl)-1,2,4-triazole (**8**)

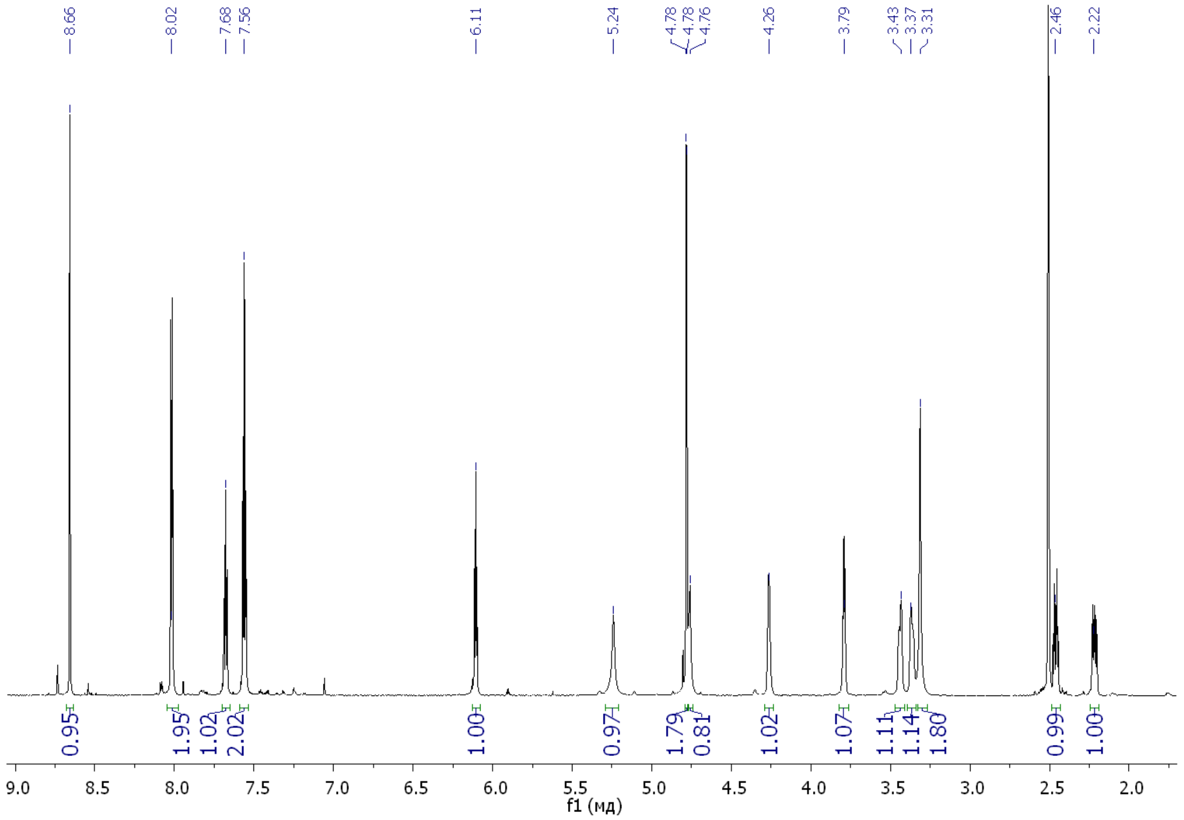

Figure S13: The ^{1}H NMR spectrum of 1-(2-deoxy-β-*D*-ribofuranosyl)-3-phenacylthio-1,2,4-triazole (**9**)

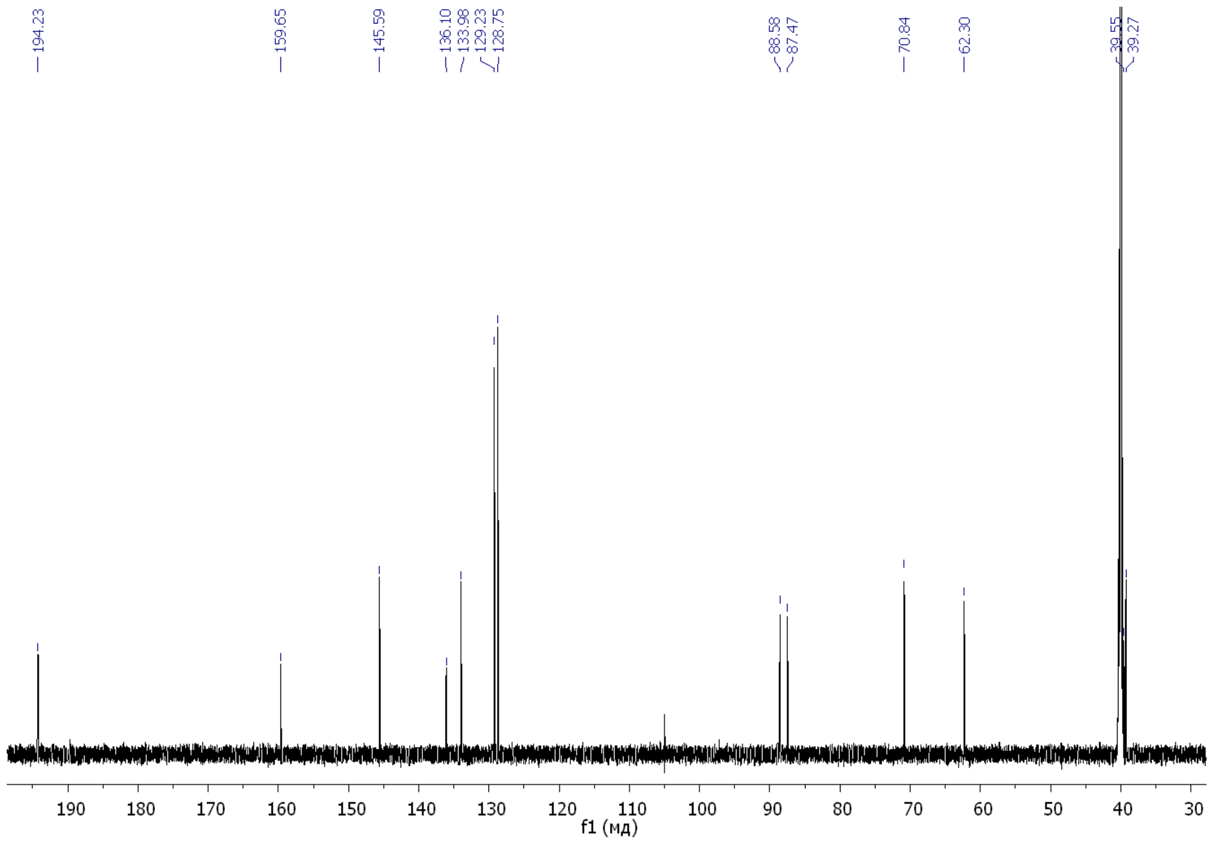

Figure S14: The ^{13}C NMR spectrum of 1-(2-deoxy-β-*D*-ribofuranosyl)-3-phenacylthio-1,2,4-triazole (**9**)

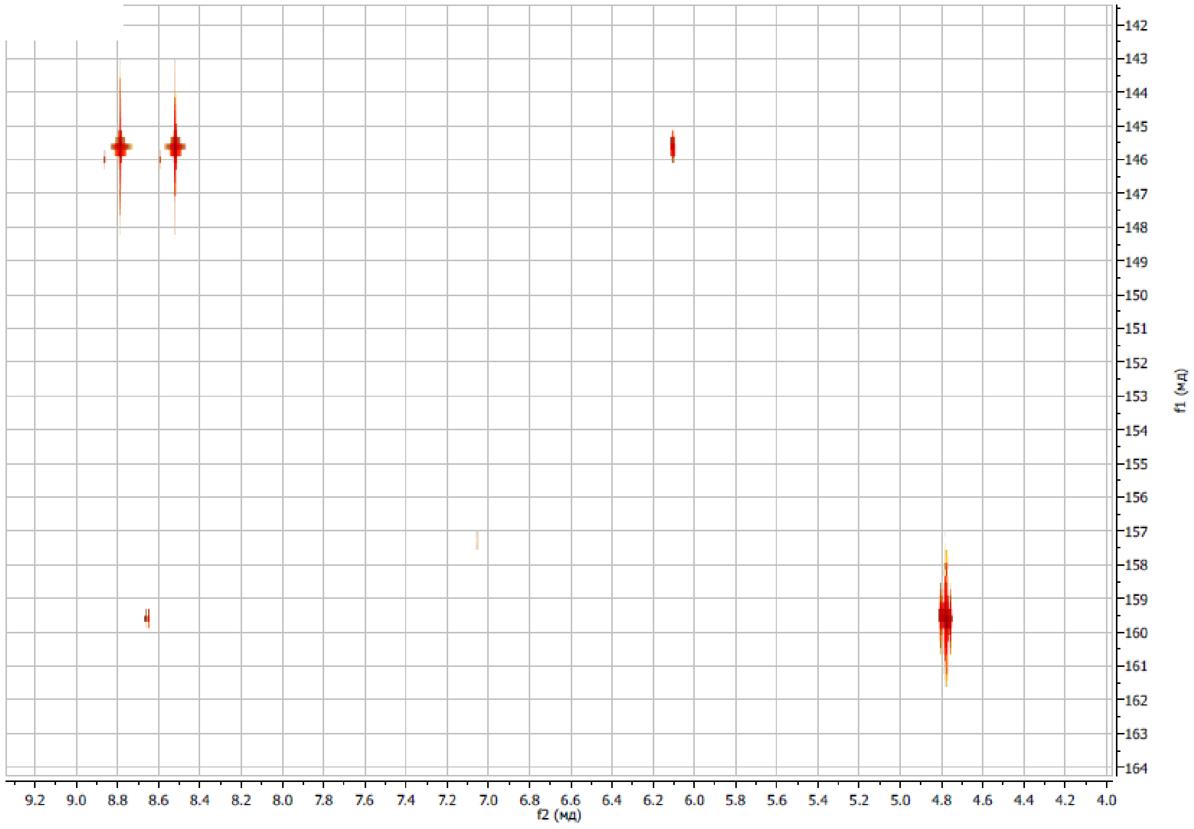

Figure S15: Fragment of the ^{1}H-^{13}C HMBC NMR spectrum of 1-(2-deoxy-β-*D*-ribofuranosyl)-3-phenacylthio-1,2,4-triazole (**9**)

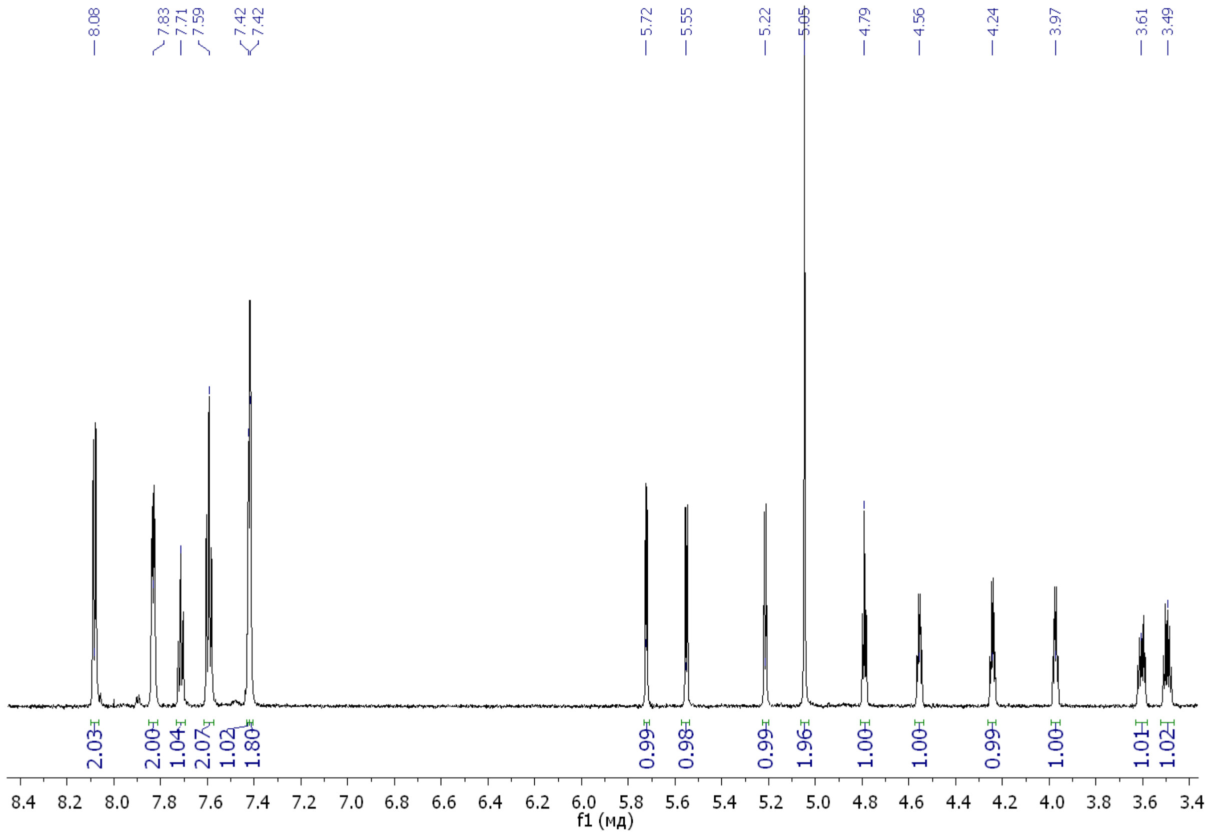

Figure S16: The ^{1}H NMR spectrum of 5-phenacylthio-3-phenyl-1-(β-*D*-ribofuranosyl)-1,2,4-triazole (**10**)

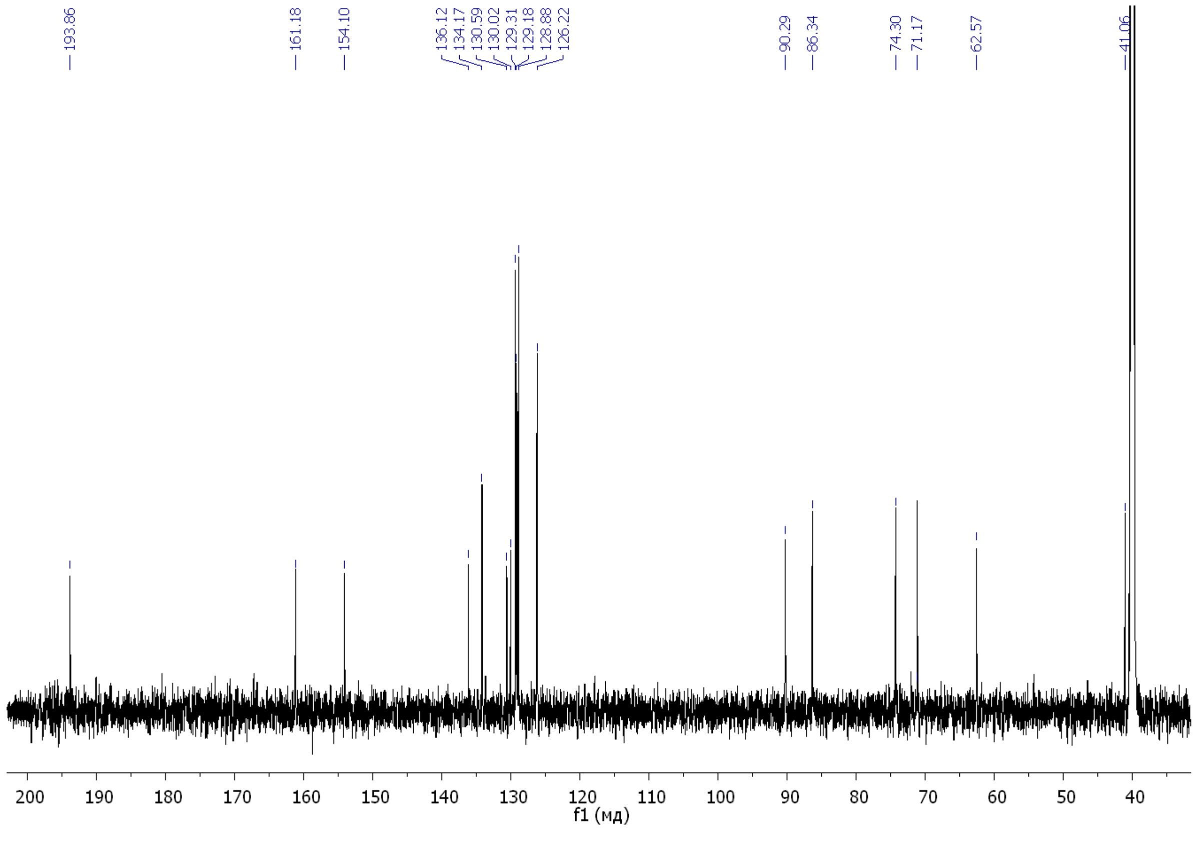

Figure S17: The ^{13}C NMR spectrum of 5-phenacylthio-3-phenyl-1-(β-*D*-ribofuranosyl)-1,2,4-triazole (**10**)

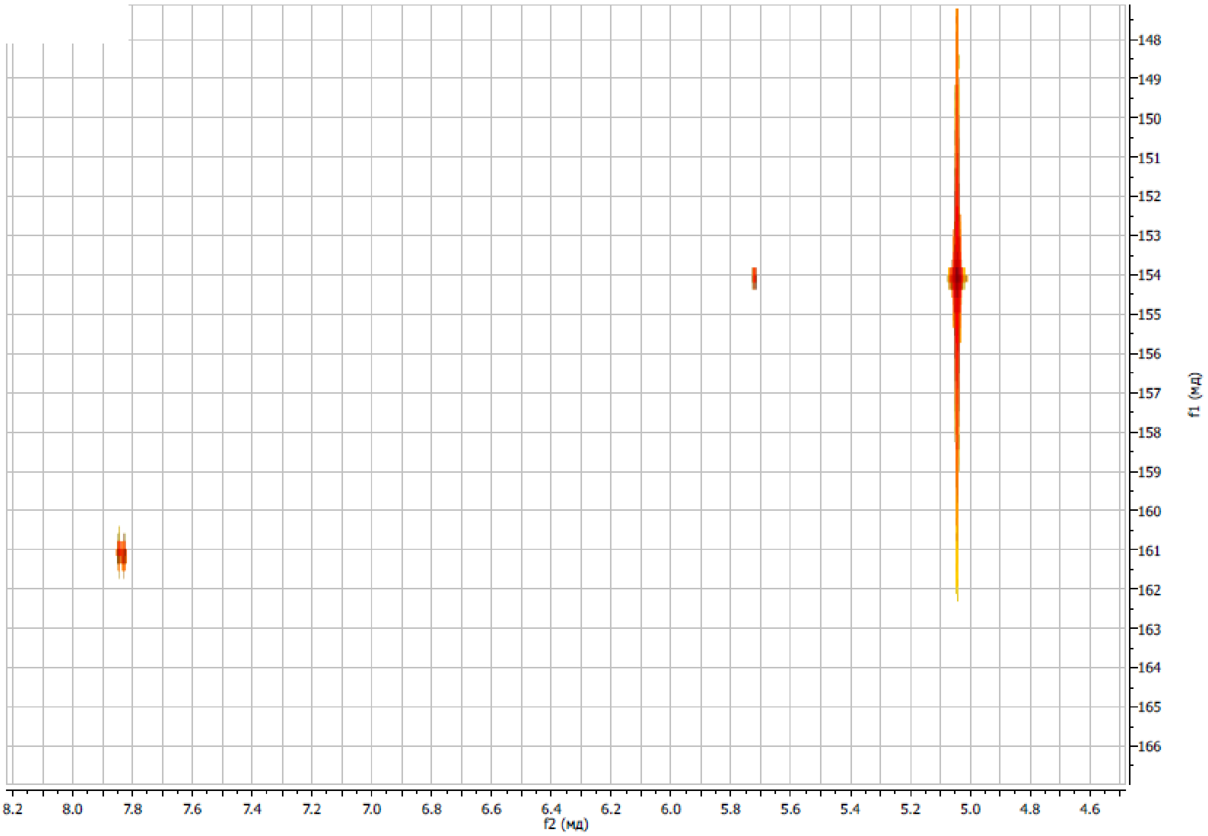

Figure S18: Fragment of the ^{1}H-^{13}C HMBC NMR spectrum of 5-phenacylthio-3-phenyl-1-(β-*D*-ribofuranosyl)-1,2,4-triazole (**10**)

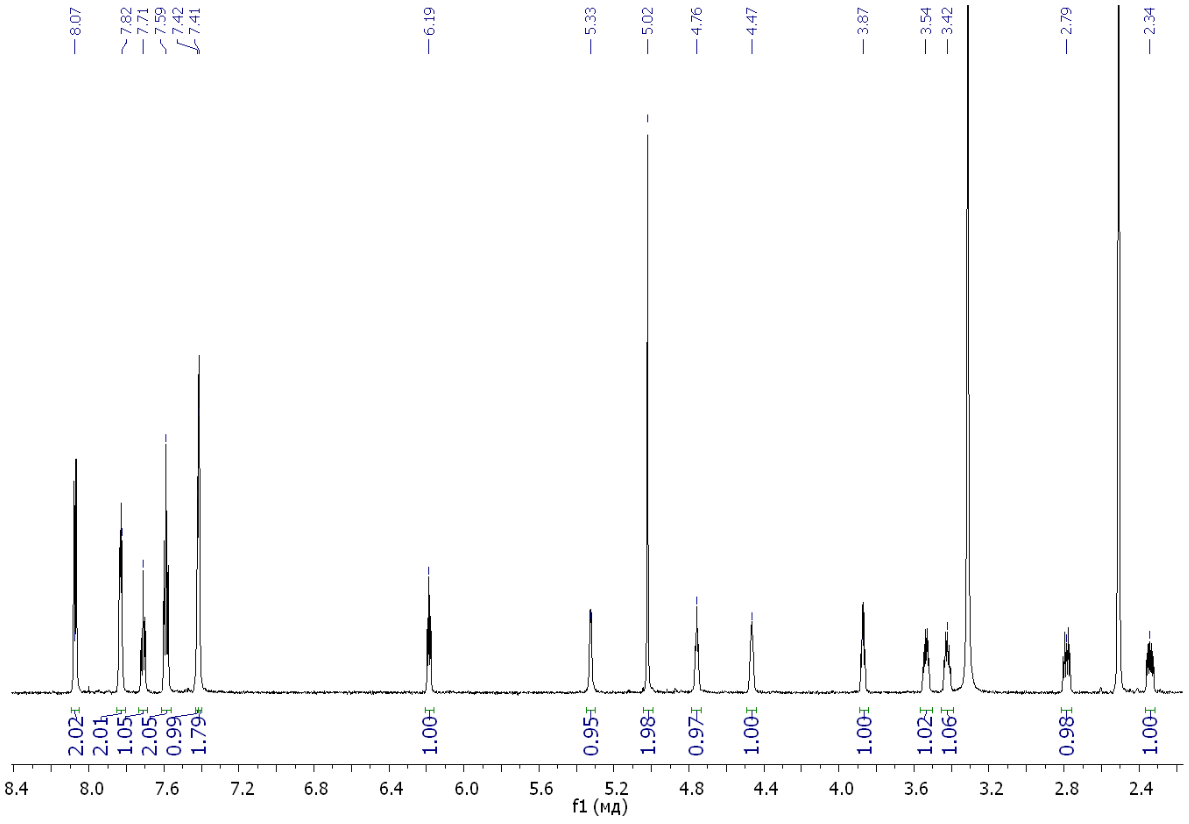

Figure S19: The ^{1}H NMR spectrum of 1-(2-deoxy-β-*D*-ribofuranosyl)-5-phenacylthio-3-phenyl-1,2,4-triazole (**11**)

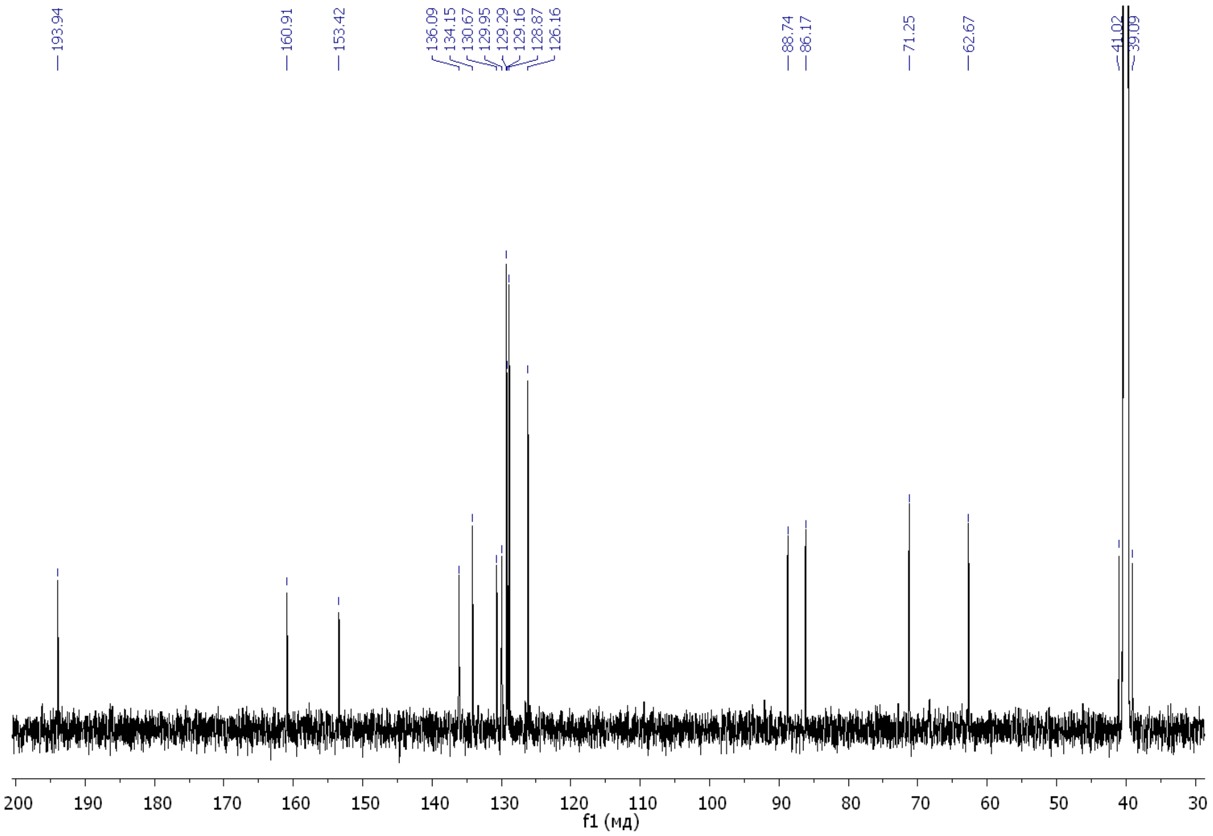

Figure S20: The ^{13}C NMR spectrum of 1-(2-deoxy-β-*D*-ribofuranosyl)-5-phenacylthio-3-phenyl-1,2,4-triazole (**11**)

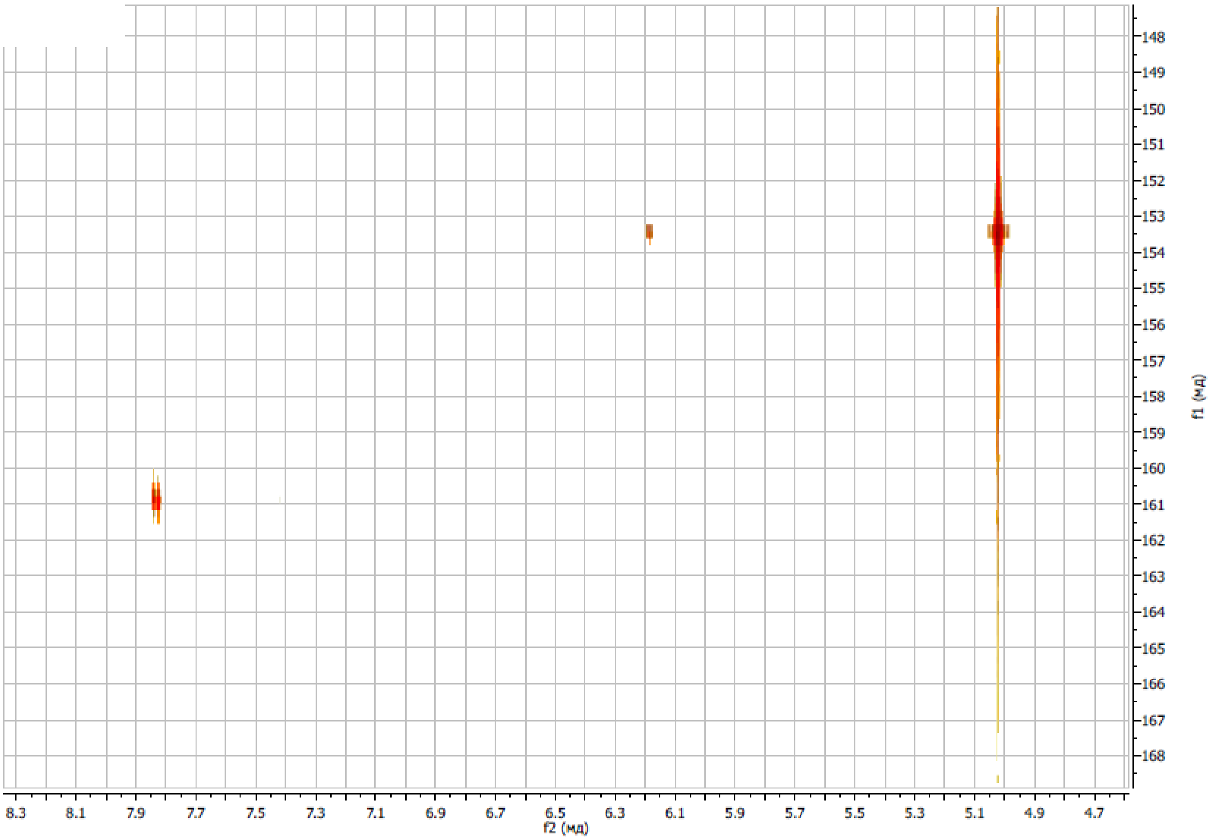

Figure S21: Fragment of the ^{1}H-^{13}C HMBC NMR spectrum of 1-(2-deoxy-β-*D*-ribofuranosyl)-5-phenacylthio-3-phenyl-1,2,4-triazole (**11**)

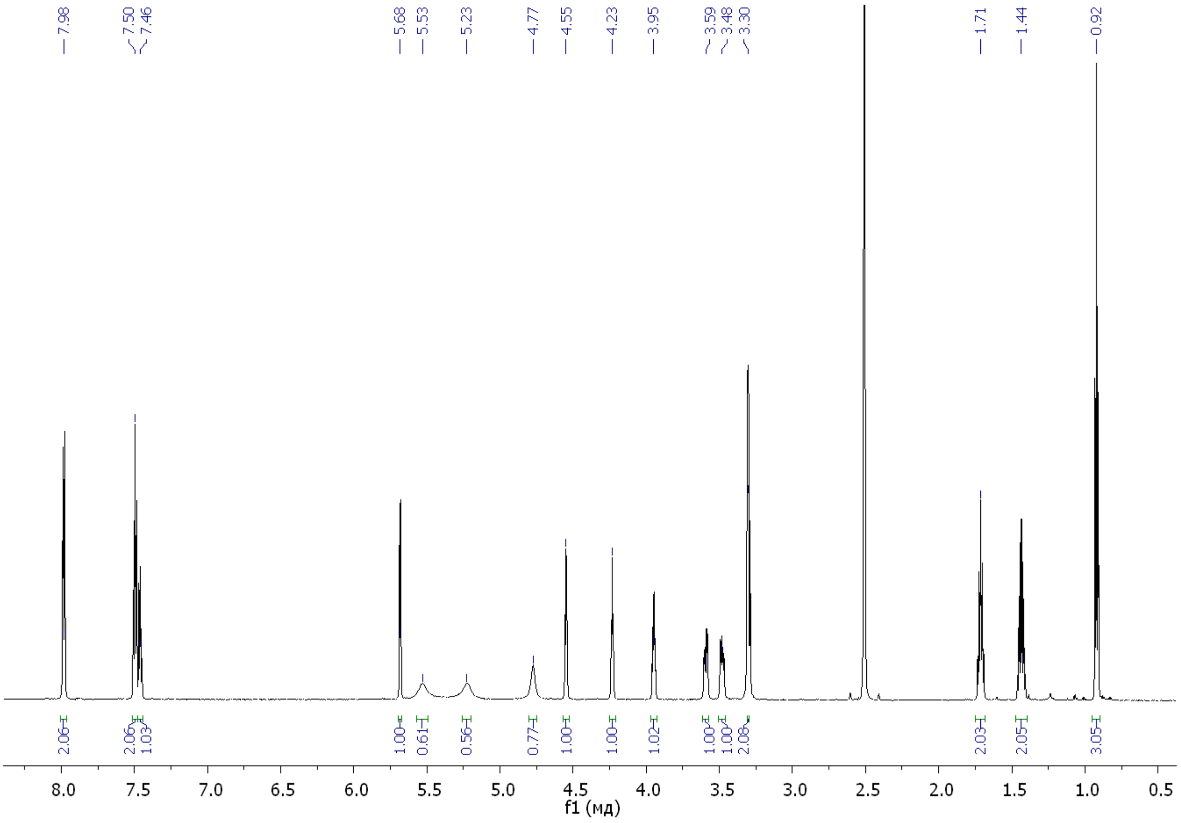

Figure S22: The ^{1}H NMR spectrum of 5-butylthio-3-phenyl-1-(β-*D*-ribofuranosyl)-1,2,4-triazole (**12**)

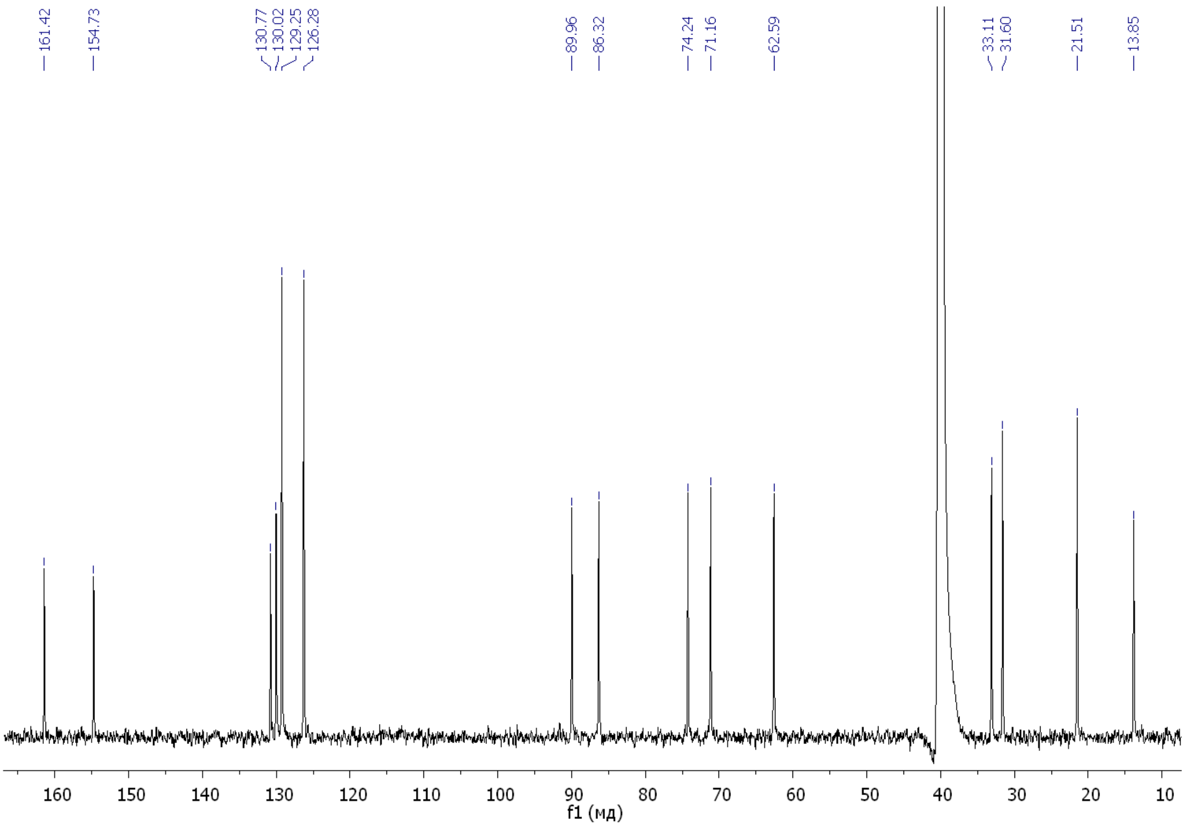

Figure S23: The ^{13}C NMR spectrum of 5-butylthio-3-phenyl-1-(β-*D*-ribofuranosyl)-1,2,4-triazole (**12**)

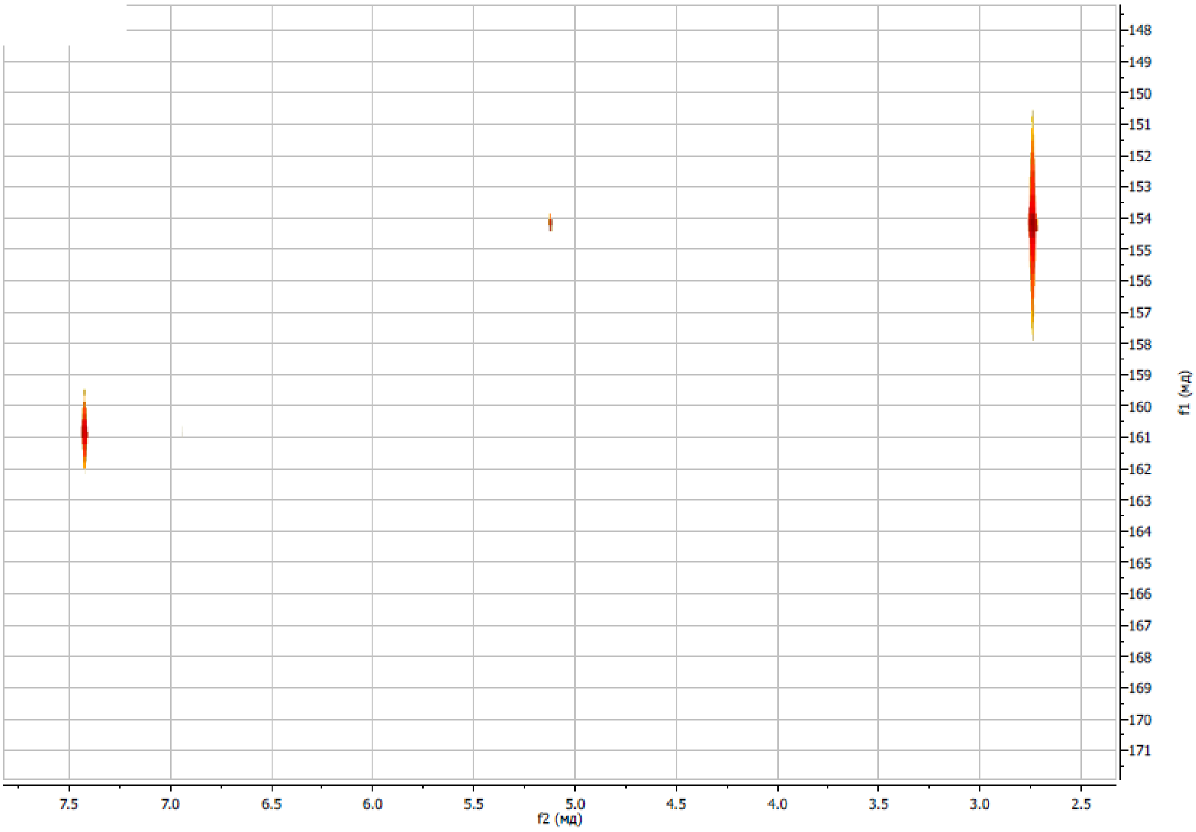

Figure S24: Fragment of the ^{1}H-^{13}C HMBC NMR spectrum of 5-butylthio-3-phenyl-1-(β-*D*-ribofuranosyl)-1,2,4-triazole (**12**)

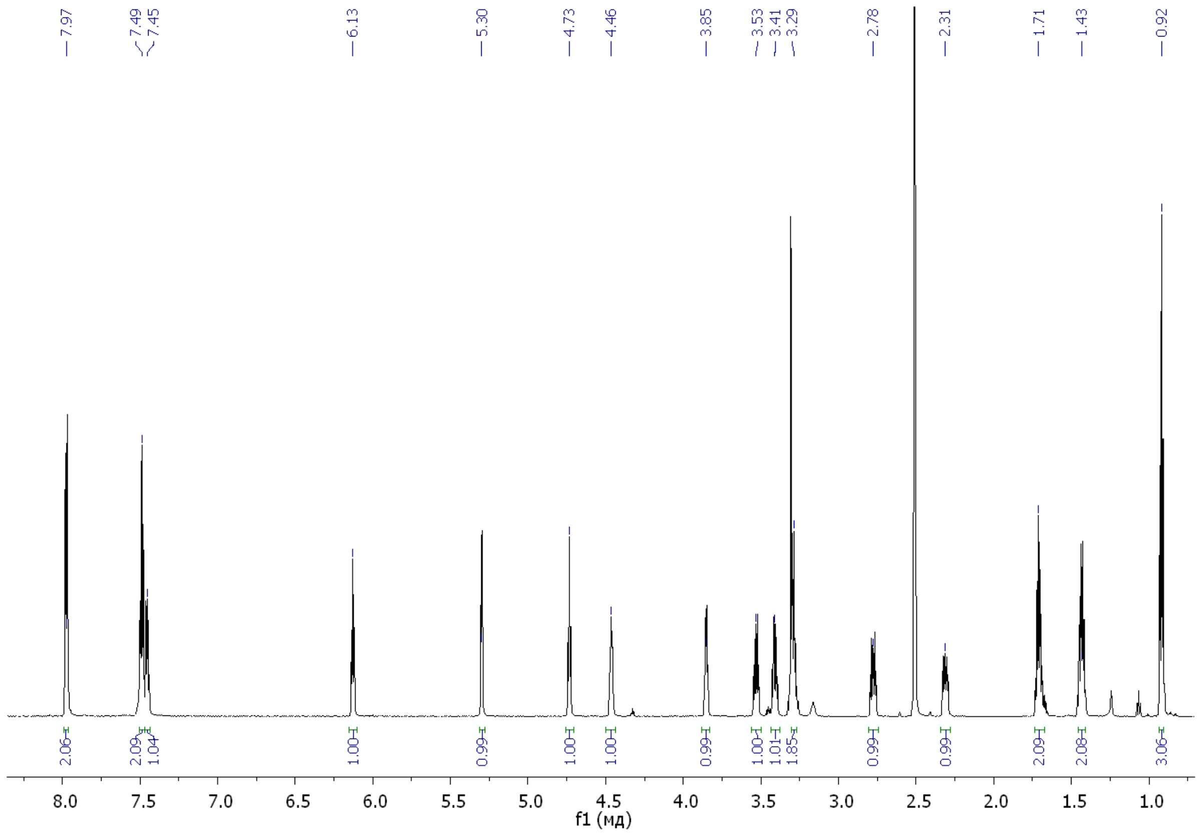

Figure S25: The ^{1}H NMR spectrum of 5-butylthio-1-(2-deoxy-β-*D*-ribofuranosyl)-3-phenyl-1,2,4-triazole (**13**)

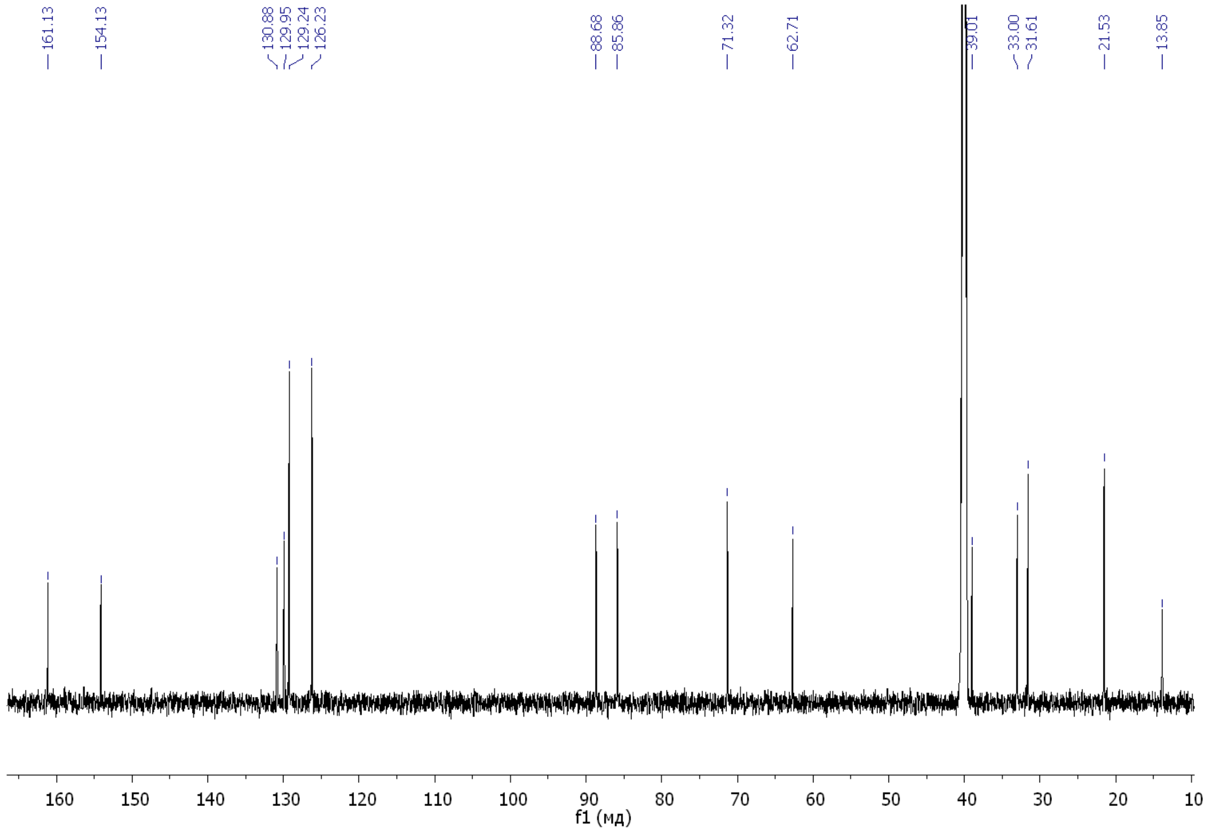

Figure S26: The ^{13}C NMR spectrum of 5-butylthio-1-(2-deoxy-β-*D*-ribofuranosyl)-3-phenyl-1,2,4-triazole (**13**)

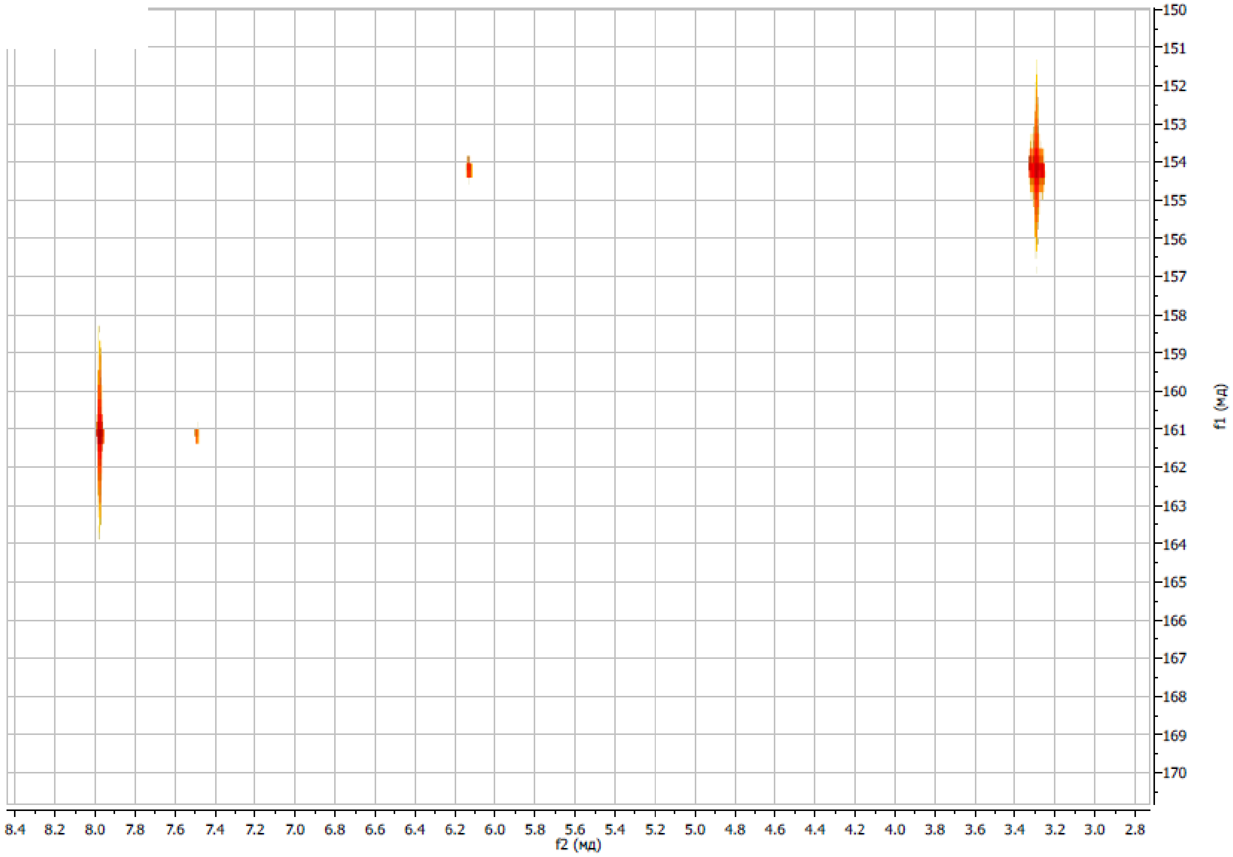

Figure S27: Fragment of the ^{1}H-^{13}C HMBC NMR spectrum of 5-butylthio-1-(2-deoxy-β-*D*-ribofuranosyl)-3-phenyl-1,2,4-triazole (**13**)
